# The long non-coding RNA *MYCNOS-01* regulates MYCN protein levels and affects growth of *MYCN*-amplified rhabdomyosarcoma and neuroblastoma cells

**DOI:** 10.1186/s12885-018-4129-8

**Published:** 2018-02-21

**Authors:** Eleanor M. O’Brien, Joanna L. Selfe, Ana Sofia Martins, Zoë S. Walters, Janet M. Shipley

**Affiliations:** Sarcoma Molecular Pathology Team, Divisions of Molecular Pathology and Cancer Therapeutics, Institute of Cancer Research, Surrey, Sutton SM2 5NG UK

**Keywords:** *MYCNOS*, *MYCN*, Rhabdomyosarcoma, Neuroblastoma, Long non-coding RNA

## Abstract

**Background:**

*MYCN* is amplified in small cell lung cancers and several pediatric tumors, including alveolar rhabdomyosarcomas and neuroblastomas. MYCN protein is known to play a key oncogenic role in both alveolar rhabdomyosarcomas and neuroblastomas. *MYCN opposite strand (MYCNOS)* is a gene located on the antisense strand to *MYCN* that encodes alternatively spliced transcripts, two of which (*MYCNOS-01* and *MYCNOS-02)* are known to be expressed in neuroblastoma and small cell lung cancer with reciprocal regulation between *MYCNOS-02* and MYCN reported for neuroblastomas. We sought to determine a functional role for *MYCNOS-01* in alveolar rhabdomyosarcoma and neuroblastoma cells and identify any associated regulatory effects between MYCN and *MYCNOS-01*.

**Methods:**

*MYCNOS-01, MYCNOS-02* and *MYCN* expression levels were assessed in alveolar rhabdomyosarcoma and neuroblastoma cell lines and tumor samples from patients using Affymetrix microarray data and quantitative RT-PCR. Following *MYCNOS-01* or *MYCN* siRNA knockdown and *MYCNOS-01* overexpression, transcript levels were assayed by quantitative RT-PCR and MYCN protein expression assessed by Western blot and immunofluorescence. Additionally, effects on cell growth, apoptosis and cell cycle profiles were determined by a metabolic assay, caspase activity and flow cytometry, respectively.

**Results:**

*MYCNOS-01* transcript levels were generally higher in NB and RMS tumor samples and cell lines with *MYCN* genomic amplification. RNA interference of *MYCNOS-01* expression did not alter *MYCN* transcript levels but decreased MYCN protein levels. Conversely, MYCN reduction increased *MYCNOS-01* transcript levels, creating a negative feedback loop on MYCN protein levels. Reduction of *MYCNOS-01* or *MYCN* expression decreased cell growth in *MYCN*-amplified alveolar rhabdomyosarcoma and neuroblastoma cell lines. This is consistent with *MYCNOS-01*-mediated regulation of MYCN contributing to the phenotype observed.

**Conclusions:**

An alternative transcript of *MYCNOS*, *MYCNOS-01*, post-transcriptionally regulates MYCN levels and affects growth in *MYCN*-amplified rhabdomyosarcoma and neuroblastoma cells.

**Electronic supplementary material:**

The online version of this article (10.1186/s12885-018-4129-8) contains supplementary material, which is available to authorized users.

## Background

Several pediatric cancers feature amplification at the chromosomal region 2p24 including alveolar rhabdomyosarcoma (ARMS), neuroblastoma (NB), medulloblastoma, Wilms’ tumor, and retinoblastoma [1–3]. The minimum common region of amplification at 2p24 in rhabdomyosarcoma (RMS) and NB has been found to consistently include the oncogene *MYCN* and amplification of *MYCN* is used clinically as a prognostic marker in NB [3–7]. Amplification or overexpression of *MYCN* leads to dysregulation of proliferation, differentiation and the cell cycle in NB [8] and contributes to cell growth in ARMS [9]. MYCN is also capable of positive auto-regulation as well as auto-suppression in NB, potentially fine-tuning MYCN levels [10–12]. In ARMS, *MYCN* transcription is driven by PAX3-FOXO1, the protein product of a fusion between the *PAX3* and *FOXO1* genes that has prognostic significance in these tumors [9, 13]. RMS and NB are a major cause of cancer related death in children with a five-year survival rate of around 50% for high-risk NB cases, including those with *MYCN* amplification, and 40% for *PAX3-FOXO1* positive RMS cases [13, 14].

*MYCN opposite-strand* (*MYCNOS, N-CYM, MYCN-AS1, NYCM, CYMN*) is produced by antisense transcription across exon 1 and intron 1 of *MYCN* that has been shown to be highly expressed in *MYCN*-amplified NB and small cell lung cancer [15, 16]. Two alternative transcripts denoted *MYCNOS-01* and *MYCNOS-02* (Additional file 1: Figure S1A) are fully sequence-verified [15]. There is an emerging body of evidence for roles of *MYCNOS-02* through an encoded protein (NCYM) that promotes NB tumorigenesis, in particular via its regulation of *MYCN* expression, and also its role as a long non-coding RNA (lncRNA) [17–22]. NCYM has been shown to mediate expression of MYCN protein by both direct interaction and also indirectly via inhibition of GSK3β, leading to decreased MYCN phosphorylation and a concomitant increase in MYCN protein stability [17]. This was associated with increased tumor growth and metastasis [17]. The same study also concluded that MYCN positively drives the promoter of *MYCNOS-02* in an E-box-dependent manner [17]. As well as increasing MYCN protein, NCYM has been found to increase MYCN cleavage to produce the anti-apoptotic protein Myc-nick in NB [22]. NCYM has also been shown to promote aggressiveness in NB by increasing *OCT4* expression via its stabilization of MYCN [20].

LncRNAs are commonly defined as transcripts of over 200 nucleotides in length that in general do not code for a protein [23]. *MYCNOS-02* lncRNA is able to regulate the usage of two *MYCN* promoters and therefore expression of different *MYCN* transcripts via interaction with binding partners such as G3BP1. This in turn results in expression of different isoforms of the MYCN protein [18]. *MYCNOS-02* lncRNA has also been found to recruit CTCF to the *MYCN* promoter to increase recruitment of activating chromatin marks and thus increase *MYCN* expression [19]. This positive regulation of *MYCN* suppressed differentiation and increased growth, invasion and metastasis in NB [19]. Additionally, a recent study has shown *MYCNOS-02* lncRNA can interact with the RNA-binding protein NonO to indirectly increase *MYCN* transcript levels post-transcriptionally [21]. Overall, these studies show that both the *MYCNOS-02* encoded protein and lncRNA play a role in growth, invasion and metastasis of NB cells [17, 19, 20].

Unlike *MYCNOS-02*, a functional role for the *MYCNOS-01* transcript has not yet been investigated, despite original annotation of its sequence being consistent with a lncRNA [15]. In this study we therefore investigated the role of *MYCNOS-01* as a lncRNA in RMS and NB. We demonstrate that *MYCNOS-01* post-transcriptionally regulates MYCN protein levels without affecting *MYCN* mRNA levels, whilst MYCN regulates *MYCNOS-01* transcription. We show that silencing of *MYCNOS-01* in RMS and NB cell lines with *MYCN* amplification reduces cell viability, similar to the effects of *MYCN* reduction. Thus, we conclude that regulation of MYCN by *MYCNOS-01* contributes to the reduction in cell growth in RMS and NB cell lines after *MYCNOS-01* silencing.

## Methods

### Translation and Kozak sequence prediction tools

Translation prediction for the *MYCNOS-01* transcript sequence was carried out using the ExPASy translate tool (http://web.expasy.org/translate/) [24] and Kozak sequence prediction was carried out using ATGpr (http://atgpr.dbcls.jp/) [25].

### Cell culture

Human ARMS cell line RMS-01 was available directly from the authors [26] and the RH30 cell line was a gift from Peter Houghton (St Jude Children’s Research Hospital, Memphis, Tennessee). The human NB cell lines KELLY and SY5Y were obtained from ECACC (cat. No. 92110411) and ATCC (cat. No. CRL-2266) respectively. RMS-01 and RH30 were cultured in DMEM (Thermo Fisher Scientific, MA, USA) and KELLY and SY5Y were cultured in RPMI-1640 medium (Thermo Fisher Scientific, MA, USA) supplemented with 10% Foetal Bovine Serum (FBS), 2 mM L-glutamine and 1% penicillin/streptomycin. The *MYCN* overexpressing and matched empty vector expressing RH30 lines were generated as previously described in Tonelli et al., (2012) [9] and cultured in DMEM supplemented with 400 μg/ml geneticin. Cells were maintained at 37 °C and 5% CO_2_. Data from short tandem repeat testing of the cell lines using the GenePrint 10 system (Promega, WI, USA) were compared with records for these cell lines in a repository database or our own archival records. This was consistent with the origin of these cell lines.

### Analyses of expression profiling data

Data uploaded to R2 Genomics Analysis and Visualisation Platform (http://r2.amc.nl) were used for analyses. These included 101 RMS samples (ITCC) [6], a set of 19 RMS cell lines (Versteeg) [27], 88 NB samples (Versteeg) and 24 NB cell lines (Versteeg) that had been previously profiled using the Affymetrix GeneChip with the HGU133 Plus2 array. Probe sets could distinguish *MYCNOS-01* and *MYCNOS-02* transcripts: probe set 216188_at detects *MYCNOS-01*, set 207028_at detects *MYCNOS-02* and set 209757_s_at was used to detect *MYCN*.

### qRT-PCR

*MYCNOS-01* and *MYCNOS-02* expression data was available from primary sample biopsies from RMS patients. Samples and details of RNA extraction were previously described [6, 28] with appropriate approvals for investigation. RNA was isolated from cell lines using the RNeasy mini kit (Qiagen, Hilden, Germany) according to the manufacturer’s instructions. Cell line cDNA was synthesised using the High Capacity cDNA Reverse Transcriptase kit (Applied Biosystems, CA, USA) and patient sample cDNA was synthesised using SuperScript II reverse transcriptase (Invitrogen, CA, USA) following the manufacturers’ protocol. Samples were run for qRT-PCR on the ViiA™ 7 Real-Time PCR System (Applied Biosystems, CA, USA). The following Taqman**®** probe and primer sets were used: *MYCNOS-01* Hs01032821_m1, *MYCNOS-02* Hs01040745_m1, *MYCN* Hs_00232074_m1. Human *ACTB* (Beta Actin) endogenous control (Applied Biosystems, CA, USA) was used to normalise gene expression. Each sample was run in triplicate. Analysis of *MYCN* expression and copy number in patient samples is described in [6].

### siRNA transfection

Oligonucleotides for specific silencing of *MYCNOS-01, MYCNOS-02* and *MYCN* were transfected into cells using Lipofectamine RNAimax (Invitrogen, CA, USA) according to the manufacturer’s instructions. All siRNAs were obtained from GE Dharmacon (CO, USA). The sequence from 5′ to 3′ for the three siRNAs against *MYCNOS-01* were as follows: siMYCNOS-01 1 GGGACAAGAGCACAGUUUCUU, siMYCNOS-01 2 GGUAAGUUAAGGUACAGCCUU, siMYCNOS-01 3 GGAGUAUUUGUUUAGUGCUUU. The sequences for the three siRNAs against *MYCNOS-02* were GAAAGAAGGGUAGUCCGAAUU for siMYCNOS-02 1, GACCGAUGCUUCUAACCCAUU for siMYCNOS-02 2, CCGCUUUGACUGCGUGUUGUU for siMYCNOS-02 3. For knockdown of MYCN a pool of three siRNAs was used with sequences GAAGAAAUCGACGUGGUCA, CCAAGGCUGUCACCACAUU, AAUUGAACACGCUCGGACU, as previously described [9]. The control siRNA used was the ON-TARGETplus non-targeting control pool (GE Dharmacon, CO, USA). Samples were analyzed by qRT-PCR, Western blot, flow cytometry or phenotypic assays at time-points indicated in the relevant figures.

### Western blotting

Protein lysates were prepared using Cell Lysis Buffer (Cell Signaling Technology, MA, USA) and their concentration measured by the Pierce**™** BCA protein assay kit (Thermo Fisher Scientific, MA, USA). Protein samples were resolved by SDS-PAGE and transferred onto PVDF membranes. Blots were incubated with the following primary antibodies: MYCN SC-791 (1:200, Santa Cruz, TX, USA), PARP 9542 (1:1000, Cell Signaling Technology, MA, USA), Phospho-C-Myc (Thr58/Ser62) 04–217 (1:4000, Merck Millipore, MA, USA), GAPDH MAB374 (1:10000, Merck Millipore, MA, USA). Blots were then incubated with rabbit (sc-2313, Santa Cruz, TX, USA) or mouse (A9044, Sigma-Aldrich, MO, USA) horseradish peroxidase-conjugated secondary antibody diluted to 1:4000 depending on primary antibody species. Blots were developed using the ECL**™** Prime Western Blotting System (GE Healthcare, IL, USA) on the Chemidoc Touch Imaging System (Bio-Rad, CA, USA). Densitometry was performed using Bio-Rad Image Lab 5.2.1 (Bio-Rad, CA, USA).

### Immunofluorescence staining

Cells cultured in chamber slides were fixed with 2% paraformaldehyde for 15 min at room temperature and permeabilised with 0.1% Triton X-100. Samples were blocked in PBS with 10% goat serum and 1% BSA for 1 h. Samples were incubated with primary MYCN antibody SC-53993 (1:500, Santa Cruz, TX, USA) overnight at 4 °C followed by secondary antibody Alexa Fluor 555 goat anti-mouse (1:400, Invitrogen, CA, USA) for 30 min at room temperature. Cells were counterstained with DAPI. Fluorescent images were captured using a Zeiss Axioplan 2 microscope (Oberkochen, Germany) using a 16× objective and a standard exposure time optimised for control treated cells. The sum of the intensity for MYCN staining was measured using Image J software and made relative to the number of cells in that field of view, indicated by DAPI.

### Plasmid production and transfection

Full-length *MYCNOS-01* transcript (RefSeq NR_110230) was cloned into the pcDNA5/TO vector (Invitrogen, CA, USA) and the construct verified by Sanger sequencing (Eurofins Genomics, Ebersberg, Germany). For plasmid transfection, Lipofectamine 2000 (Invitrogen, CA, USA) was used following the manufacturer’s instructions. Samples were analyzed by qRT-PCR and Western blot at time-points indicated in the relevant figures.

### Proteasome inhibition

For protein stability experiments, cells were transfected for a total of 48 h and treated for the final 4 h with either 10 μM MG132 (Sigma-Aldrich, MO, USA) in DMSO or DMSO control. Protein was then extracted from cells for analysis by Western blot.

### Cell viability assay

Cells were transfected as six repeats in a 96-well plate to assess the effects of gene knockdown. Cell viability was assessed by the MTS method using the CellTiter 96**®** Aqueous One Solution Cell Proliferation Assay (Promega, WI, USA). Fresh media plus 20 μl assay reagent were added at the indicated time-point. After 2.5 h incubation at 37 °C and 5% CO_2_ the absorbance of each well was measured at 492 nm on a FLUOstar Optima plate reader (BMG Labtech, Ortenberg, Germany).

### Apoptosis assay

Cells were transfected in quadruplicate in a 96-well plate and apoptosis measured by evaluating the activation of caspase 3/7 at the indicated time-point by replacement of 50 μl of media with 50 μl of Caspase-Glo**®** 3/7 Assay (Promega, WI, USA). After 1 h incubation protected from light at room temperature, the samples were transferred to a white-walled 96-well plate and luminescence of each well was read on a FLUOstar Optima plate reader (BMG Labtech, Ortenberg, Germany). The caspase signal intensity was normalised by the absorbance measurement from the corresponding MTS assay.

### Flow cytometry

Cells were fixed in 70% ethanol at -20 °C overnight, washed with PBS and resuspended in 1 ml PBS containing 100 μg/ml RNase A and 40 μg/ml Propidium Iodide. Samples were incubated for 30 min at 37 °C then analyzed on a BD™ LSRII Flow Cytometer (BD Biosciences, CA, USA).

### Statistical analysis

Graphs represent means ± standard deviation from multiple independent experiments as stated in figure legends. Statistical significance was measured by unpaired two-tailed Student *t*-test or by one-way analysis of variance (ANOVA) with post hoc Dunnett’s test for multiple comparisons. For linear correlation studies of gene expression Pearson’s coefficient (*R*) was calculated between each pair of variables to indicate the strength of the linear association. *p* < 0.05 was considered significant and indicated by a single asterisk, *p* < 0.01 is indicated by a double asterisk, and *p* < 0.001 is indicated by a triple asterisk.

## Results

### *MYCNOS-01* is predicted to be non-protein coding and correlations between *MYCNOS* and *MYCN* transcript levels in RMS and NB patients and cell lines

**Fig. 1:**
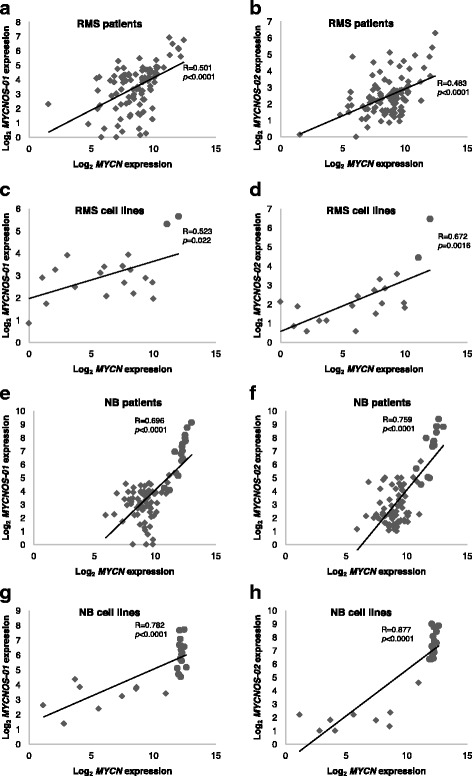
Correlation of *MYCNOS* transcripts and *MYCN* transcript expression in RMS and NB. **a** Correlation between *MYCNOS-01* and *MYCN* measured by Affymetrix microarray in 101 RMS patient samples (*R* = 0.501, *p* < 0.0001) and (**b**) correlation between *MYCNOS-02* and *MYCN* in these patient samples (*R* = 0.483, *p* < 0.0001). **c** Correlation between *MYCNOS-01* and *MYCN* measured by Affymetrix microarray in 19 RMS cell lines (*R* = 0.523, *p* = 0.022) and (**d**) correlation between *MYCNOS-02* and *MYCN* (*R* = 0.672, *p* = 0.0016). **e** Correlation between *MYCNOS-01* and *MYCN* measured by Affymetrix microarray in 88 NB patient samples (*R* = 0.696, *p* < 0.0001) and (**f**) correlation between *MYCNOS-02* and *MYCN* in these patient samples (*R* = 0.759, *p* < 0.0001). **g** Correlation between *MYCNOS-01* and *MYCN* measured by Affymetrix microarray in 24 NB cell lines (*R* = 0.782, *p* < 0.0001) and (**h**) correlation between *MYCNOS-02* and *MYCN* (*R* = 0.877, *p* < 0.0001). Data derived from R2 genomics analysis and visualisation platform. *MYCN*-amplified cases or cell lines are depicted with circles

Previous data showed protein encoding potential for *MYCNOS-02* transcripts [15]. In contrast, translation and Kozak sequence analysis for *MYCNOS-01* predicted transcripts to be non-protein coding since the probability any start codon present in the *MYCNOS-01* sequence is an initiation codon is very low (Additional file 1: Figure S1B and C). The relationship between *MYCN* and *MYCNOS-01* transcript levels was then investigated by mining publically available expression profiling data for RMS and NB samples from patients and cell lines. A significant correlation between levels of *MYCNOS-01* and *MYCN* expression and *MYCNOS-02* and *MYCN* expression was identified considering all RMS samples (Fig. 1a and b) and cell lines (Fig. 1c and d). There was also a significant positive correlation between both *MYCNOS* transcripts and *MYCN* transcript expression in NB patient samples (Fig. 1e and f) and cell lines (Fig. 1g and h) that is consistent with the literature for *MYCNOS-02* [17, 19]. For NB patient samples and cell lines, levels of *MYCNOS-01* and *02* were higher in cases with *MYCN* amplification (Fig. 1e-h) versus cases without. Amplification at 2p24 in RMS is also associated with high *MYCNOS-01* and *MYCNOS-02* expression levels, as indicated in data mined for the cell lines (Fig. 1c and d) and qRT-PCR analyses of a limited number of cases with known *MYCN* amplification status as well as the cell lines (Additional file 2: Figure S2A–D). *MYCN* and *MYCNOS-01* transcript levels in RMS and NB showed no significant correlations when *MYCN* amplified cases were excluded (RMS *p* = 0.72, R = 0.078; NB *p* = 0.42, R = 0.097).

### *MYCNOS-01* regulates MYCN protein but not transcript levels in *MYCN*-amplified RMS and NB cells

To determine whether *MYCNOS-01* regulates *MYCN* transcript levels, which may be consistent with the correlations in their expression levels in NB and RMS derived samples, we performed siRNA-mediated silencing of *MYCNOS-01* in cell lines. RMS cell lines tested had either high (RMS-01) or intermediate (RH30) MYCN expression levels, and NB cell lines used had high (KELLY) and low (SY5Y) levels of MYCN [7–9]. The relative *MYCNOS* and *MYCN* transcript levels for the four cell lines used are indicated in Additional file 2: Figure S2E.

**Fig. 2:**
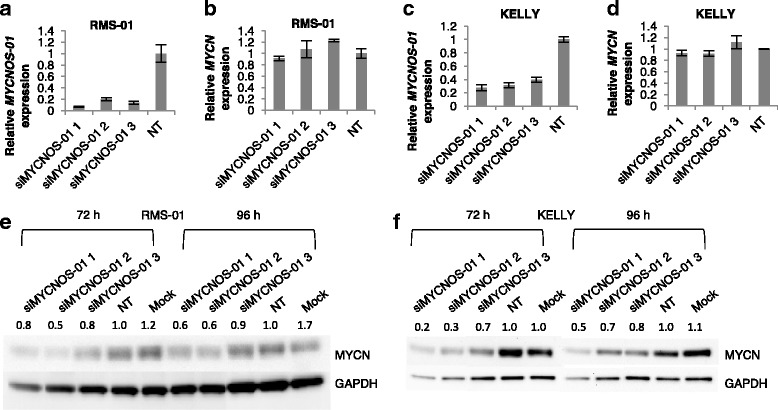
*MYCN* transcript and protein expression after *MYCNOS-01* knockdown in high *MYCN*-expressing RMS and NB. qRT-PCR detecting *MYCNOS-01* transcript level after *MYCNOS-01* knockdown for 72 h with three siRNAs in (**a**) RMS-01 and (**c**) KELLY. Corresponding *MYCN* transcript level shown in (**b**) RMS-01 and (**d**) KELLY. Expression relative to NT control. (**e**) and (**f**) MYCN protein levels after *MYCNOS-01* knockdown for 72 h and 96 h with three siRNAs in RMS-01 and KELLY respectively. GAPDH used as loading control. Densitometry values shown above each blot normalised to GAPDH and relative to NT control. Data representative of three repeats. NT = non-targeting control. Relative expression of transcripts in these cell lines are indicated in Additional file 2: Figure S2E

**Fig. 3:**
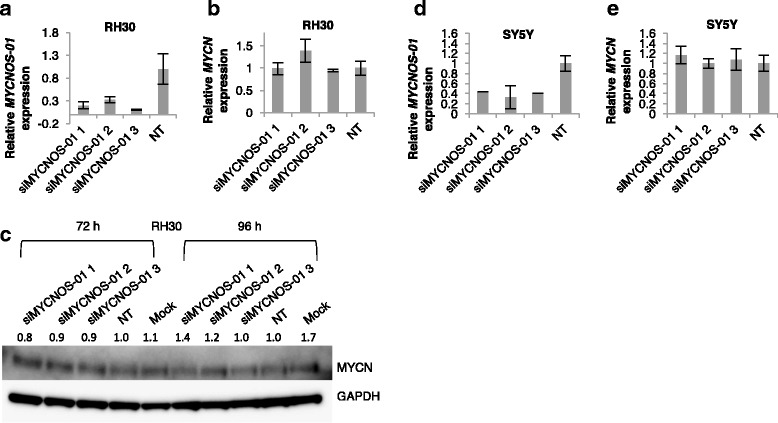
*MYCN* transcript and protein expression after *MYCNOS-01* knockdown in intermediate and low *MYCN*-expressing RMS and NB. qRT-PCR detecting *MYCNOS-01* transcript level after *MYCNOS-01* knockdown for 72 h with three siRNAs in (**a**) RH30 and (**d**) SY5Y. Corresponding *MYCN* transcript level shown in (**b**) RH30 and (**e**) SY5Y. Expression relative to NT control. (**c**) MYCN protein levels after *MYCNOS-01* knockdown for 72 h and 96 h with three siRNAs in RH30. GAPDH used as loading control. Densitometry values shown above each blot normalised to GAPDH and relative to NT control. Data representative of three repeats. NT = non-targeting control. Relative expression of transcripts in these cell lines are indicated in Additional file 2: Figure S2E

In high *MYCN*-expressing lines, no consistent effect on *MYCN* transcript levels was observed after silencing of *MYCNOS-01* for 72 h (Fig. 2a-d). To determine whether *MYCNOS-01* is regulating MYCN post-transcriptionally, we also assessed MYCN protein levels by Western blotting. MYCN protein was decreased in cells transfected with *MYCNOS-01* siRNAs compared to non-targeting control in high *MYCN*-expressing cell lines RMS-01 and KELLY (Fig. 2e, f). This is supported by the decrease in MYCN immunofluorescence signal after *MYCNOS-01* depletion compared to control in these cell lines (Additional file 3: Figure S3A-F). Furthermore, *MYCNOS-01* overexpression has no effect on *MYCN* transcript levels but causes a slight increase in MYCN protein; the effect observed is limited since RMS-01 and KELLY cells express very high basal levels of MYCN (Additional file 4: Figure S4). Experiments on RMS-01 cells including a proteasome inhibitor showed no increase in phosphorylated MYCN with *MYCNOS-01* knockdown, indicating that MYCN protein stability was not affected (Additional file 5: Figure S5). However, *MYCNOS-01* silencing had no strong effect on either *MYCN* transcript or protein levels in the ARMS cell line RH30 with intermediate *MYCN* expression (Fig. 3a-c). Due to the low expression levels of MYCN protein in the NB cell line SY5Y, no bands were visible by Western blot but there was similarly no *MYCNOS-01*-mediated effect on *MYCN* transcript expression (Fig. 3d and e).

As a role for *MYCNOS-02* in RMS has not been previously evaluated, we also performed siRNA-mediated silencing of *MYCNOS-02* to determine the effects on *MYCN* mRNA and protein expression in RMS-01 and KELLY cells (Additional file 6: Figure S6). Similar levels of *MYCNOS-02* silencing were observed with all 3 siRNAs, but there was no consistent effect on *MYCN* expression at the RNA or protein level.

Overall, these results demonstrate that *MYCNOS-01* reduces MYCN protein levels in *MYCN*-amplified RMS and NB cell lines but not through altering MYCN phosphorylation.

### MYCN regulates *MYCNOS-01* transcript levels

**Fig. 4:**
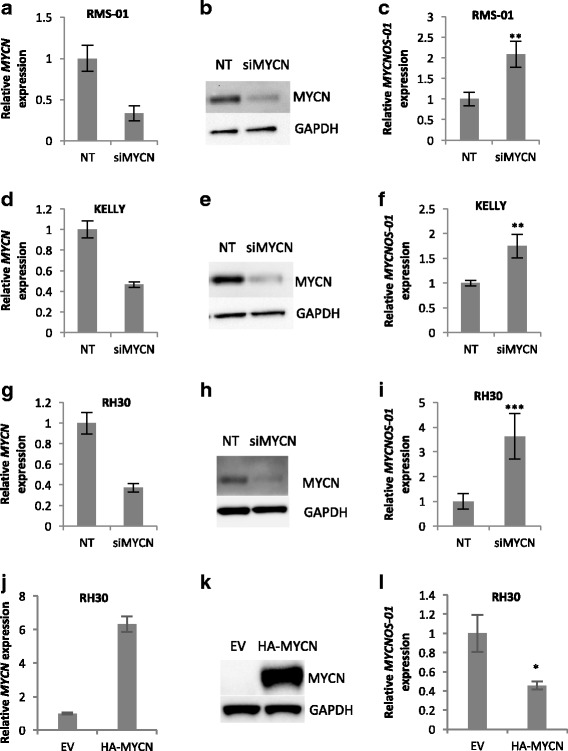
Effect of *MYCN* knockdown on *MYCNOS-01* transcript expression in RMS and NB. **a** qRT-PCR and (**b**) Western blot for MYCN, and (**c**) qRT-PCR for *MYCNOS-01* after treatment with three pooled *MYCN* siRNAs for 72 h in RMS-01. **d-f** in KELLY as for (**a**)-(**c**). **g-i** in RH30 as for (**a**)-(**c**). **j** qRT-PCR and (**k**) Western bot for MYCN, and (**l**) qRT-PCR for *MYCNOS-01* in RH30 stably transfected with empty vector (EV) or HA-MYCN expressing vector. Graphs and Western blots representative of three repeats. NT = non-targeting control

Silencing *MYCN* in RMS and NB caused a significant increase in *MYCNOS-01* expression in both high *MYCN*-expressing (RMS-01, KELLY) and intermediate *MYCN*-expressing (RH30) RMS and NB cell lines (Fig. 4a-i). Conversely, overexpressing *MYCN* in RH30 decreased *MYCNOS-01* expression (Fig. 4j-l). MYCN was found to similarly regulate *MYCNOS-02* in RMS and NB with *MYCN* amplification (Additional file 7: Figure S7). Overall, these results indicate MYCN negatively regulates *MYCNOS-01* expression whilst *MYCNOS-01* positively regulates MYCN protein levels.

### Decreasing *MYCNOS-01* levels results in decreased cell viability in *MYCN*-amplified RMS and NB cells

**Fig. 5:**
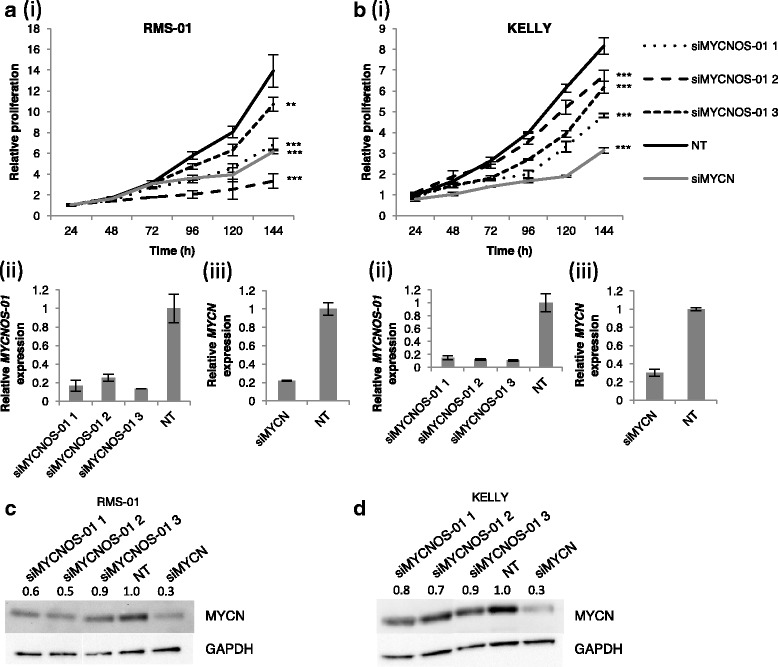
Knockdown of *MYCNOS-01* inhibits cell viability in high *MYCN*-expressing RMS and NB. (**a***i*) MTS assay showing cell viability of RMS-01 up to 144 h after transfection with three *MYCNOS-01* siRNAs or three pooled *MYCN* siRNAs relative to NT siRNA. (**b***i*) KELLY as in (**a***i*). Corresponding qRT-PCR for *MYCNOS-01* (*ii*) and *MYCN* (*iii*) at 24 h shown below line graph. **c** MYCN protein levels after *MYCNOS-01* or *MYCN* knockdown for 72 h in RMS-01 and (**d**) in KELLY. Densitometry values shown above each blot normalised to GAPDH and relative to NT control. Data representative of three repeats. Statistical analysis relative to non-targeting control. NT = non-targeting

**Fig. 6:**
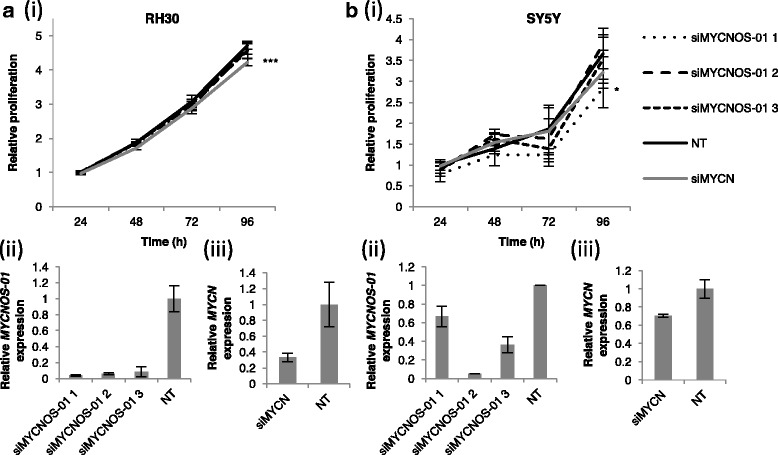
Knockdown of *MYCNOS-01* does not inhibit cell viability in intermediate or low *MYCN*-expressing RMS and NB. (**a**i) MTS assay showing cell viability of RH30 up to 96 h after transfection with three *MYCNOS-01* siRNAs or three pooled *MYCN* siRNAs relative to NT siRNA. (**b**i) SY5Y as in (**a**i). Corresponding qRT-PCR for *MYCNOS-01* (*ii*) and *MYCN* (iii) at 24 h shown below line graph. Data representative of three repeats. Statistical analysis relative to non-targeting control. NT = non-targeting

**Fig. 7:**
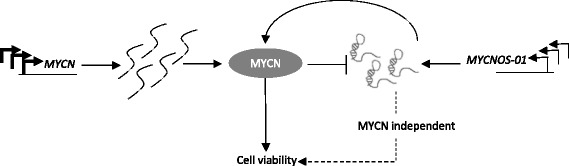
Schematic of proposed model for regulation between MYCN and *MYCNOS-01*. *MYCN* and *MYCNOS-01* expression are both driven by genomic amplification, although *MYCN* is expressed at higher levels than *MYCNOS-01*. *MYCNOS-01* positively regulates MYCN protein levels and MYCN negatively regulates *MYCNOS-01* transcript levels as a form of negative feedback on MYCN. *MYCNOS-01*-mediated regulation of MYCN has effects on cell viability but there is also likely a MYCN-independent signaling pathway

We next investigated the phenotypic effects of *MYCNOS-01* and *MYCNOS-02* depletion on RMS (RMS-01, RH30) and NB (KELLY, SY5Y) cells. Decreasing *MYCNOS-01* expression resulted in a significant decrease in cell viability compared to negative control with all *MYCNOS-01* siRNAs in *MYCN*-amplified RMS-01 and KELLY cells (Fig. 5a and b), similar to the reduction in cell viability that is seen with siRNA-mediated *MYCN* silencing (Fig. 5a and b). Silencing *MYCNOS-01* did not reduce MYCN protein lower than direct *MYCN* silencing (Fig. 5c), but showed a similar or greater effect on RMS cell viability (Fig. 5a) raising the possibility that *MYCNOS-01* may have targets in addition to MYCN in RMS. Decreasing *MYCNOS-02* expression also significantly decreased cell viability in these cell lines (Additional file 8: Figure S8A and B). In contrast, silencing of *MYCNOS-01* did not significantly affect cell viability in intermediate (RH30) and low (SY5Y) *MYCN* expressing cells overall (Fig. 6a and b). However, although the effect was less for RH30 compared to RMS-01, *MYCN* knockdown did significantly decrease cell growth as we have previously reported [9] (Fig. 6a). *MYCN* reduction in SY5Y had no effect, although levels of MYCN are very low (Fig. 6b). There was no significant increase in caspase 3/7 activation or PARP cleavage in *MYCNOS-01* or *MYCN* siRNA treated cells compared to non-targeting control indicating apoptosis was not induced (Additional file 9: Figure S9). However, *MYCNOS-02* knockdown promoted apoptosis in RMS-01 and KELLY cells (Additional file 10: Figure S10). Decreasing *MYCNOS-01* expression had no effect on cell cycle progression but silencing *MYCN* caused a G1 arrest in both RMS-01 and KELLY (Additional file 11: Figure S11). Based on all our results, we propose a feedback model for *MYCNOS-01* and MYCN regulation in RMS and NB (Fig. 7).

## Discussion

In this study, we have shown that *MYCNOS-01* and *MYCNOS-02* can play important roles in RMS as well as NB cell growth, at least in part via their regulation of MYCN. Roles for *MYCNOS-01* in RMS and NB and *MYCNOS-02* in RMS have not been previously explored whilst our data for the effect of *MYCNOS-02* on growth of NB cells is consistent with previous findings [17–22]. Regulation of MYCN by *MYCNOS-01* and *MYCNOS-02* was readily apparent in *MYCN-*amplified RMS and NB, which express these transcripts at high levels, presumably as a result of their co-amplification at the genomic level. In contrast, effects of *MYCNOS* transcripts on MYCN protein levels in RMS and NB without high level *MYCN* amplification were either less marked or not seen. The positive regulation of MYCN by *MYCNOS-01* and *MYCNOS-02* likely contribute to the cell growth of RMS and NB. This is consistent with the phenotypic dose dependent effects and dependencies of RMS and NB cells on MYCN levels seen in this and previous studies [9, 29, 30].

In addition to the positive regulation of MYCN by *MYCNOS-01*, MYCN also negatively regulates *MYCNOS-01* transcription, summarized in Fig. 7. As MYCN protein has been found to be recruited to its own intron 1 [10], in line with the transcription start site for *MYCNOS-01* on the opposite strand, this binding activity could be involved in MYCN-mediated regulation of *MYCNOS-01*. Previous studies have also identified indirect MYCN negative feedback mechanisms involving *trans*-acting factors [12]. This is likely a mechanism that fine-tunes MYCN expression levels.

An increasing number of lncRNAs have now been characterized and many have been linked to cancer progression [31]. Here we have shown that *MYCNOS-01* can act as a *cis*-antisense lncRNA on its sense partner MYCN. Although *MYCN* transcript expression was not regulated by *MYCNOS-01*, we have identified a post-transcriptional role for this lncRNA in regulating MYCN protein levels. This is consistent with the lack of correlation we found between the two transcripts in non-amplified lines.

There are several examples of lncRNAs that regulate protein partners post-transcriptionally without affecting transcript expression [32–35]. For example, the lncRNA *PVT1* has been shown to be required for increasing MYC protein stability and high expression levels in 8q24-amplified cancers [32]. In our study, we found no evidence that *MYCNOS-01* altered the stability of MYCN protein via Thr58/Ser62 phosphorylation. Another example of post-transcriptional regulation is the lncRNA *treRNA*, which has been shown to play a role in tumor invasion and metastasis in breast cancer, and which regulates the translation of E-cadherin mRNA in these cells via redistribution of *CDH1* to low molecular weight polysomes to suppress translation [34]. Potentially *MYCNOS-01* could have a similar mechanism to regulate translation efficiency of MYCN and thus its protein expression. In addition, the lncRNA *BACE1-AS* regulates translation of *BACE1* by masking the binding site for miR-485-5p*,* thus preventing miRNA-induced translational repression and mRNA decay [35]. Another possibility therefore is that *MYCNOS-01* interacts with an miRNA that targets MYCN for degradation, therefore increasing MYCN protein expression by sequestering away a negative regulating factor. The above examples indicate possible post-transcriptional mechanisms that could be explored for *MYCNOS-01*-mediated regulation of MYCN. Further investigations are required to determine molecular interactions with *MYCNOS-01* and how these regulate MYCN protein levels.

Both *MYCNOS-01* and *MYCNOS-02* shown in this study in RMS and NB, and *MYCNOS-02* shown previously in NB, regulate MYCN protein levels [17, 19, 20]. However, data on whether *MYCNOS-02* is able to regulate MYCN at the transcriptional level is conflicting. One report for *MYCNOS-02* indicates silencing of *MYCNOS-02* does not affect *MYCN* expression at the transcriptional level [17]. However, other studies suggest that *MYCNOS-02* can affect *MYCN* transcript expression in NB [18, 19, 21]. *MYCNOS-02* has been found to interact with CTCF to affect chromatin remodeling at the *MYCN* promoter therefore *MYCN* transcript expression [19]. However, in silico prediction techniques suggest CTCF interacts with a region of *MYCNOS-02* that does not overlap with the sequence of *MYCNOS-01*, supporting the possibility that the two transcripts could have different binding partners that are involved in MYCN regulation. It is possible for two overlapping lncRNAs from the same locus to have different characteristics and function. For example, the *CCAT1* locus encodes *CCAT1-L* that is located in the nucleus and positively regulates *MYC* transcription and *CCAT1-S* that is mainly located in the cytoplasm with no effect on *MYC* transcript levels [36–38].

Previous studies of *MYCNOS-02* have identified its role in NB tumor growth and metastasis. In vitro*, MYCNOS-02* can suppress differentiation and promote metastasis, invasion and cell proliferation partially due to its indirect regulation of MYCN [19]. Our study found *MYCNOS-01* also plays a role in *MYCN*-amplified RMS and NB cell viability, although no effect was seen on cell cycle progression. Often a decrease in proliferation occurs with concomitant cell cycle arrest, however it is possible for these two effects to be separated. For example, one study found decreasing the tumor suppressors RPL5 or RPL11 resulted in a reduction in ribosome content and translation capacity, causing cells to progress at a lower rate through all stages of the cell cycle thus resulting in decreased proliferation without cell cycle arrest [39].

*MYCNOS-01* was also found to play a role in cell growth via regulation of MYCN; silencing of *MYCNOS-01* resulted in a reduction in MYCN protein levels. However, *MYCNOS-01* knockdown did produce a slightly different phenotype to *MYCN* knockdown due to differences in MYCN protein levels achieved. We have previously identified that using different molecular tools to diminish MYCN can affect the strength of phenotype detected depending on the magnitude and endurance of MYCN reduction [9]. *MYCNOS-01* knockdown did not decrease MYCN protein sufficiently to produce a G1 arrest, in contrast to direct *MYCN* knockdown. However, *MYCNOS-01* reduction affecting other protein targets and signaling pathways that contribute to the phenotype observed cannot be excluded. Further defining how *MYCNOS-01* regulates MYCN, and possibly other proteins, may lead to new approaches to perturb the clinically aggressive phenotype of RMS and NB tumors.

## Conclusions

*MYCNOS-01* can be added to the growing list of lncRNAs involved in tumorigenesis; *MYCNOS-01* positively regulates MYCN and MYCN negatively regulates *MYCNOS-01,* potentially to fine-tune MYCN protein levels. *MYCNOS-01* affects cell growth of *MYCN*-amplified RMS and NB and could also play a role in other *MYCN*-driven cancers. Although directly targeting lncRNAs and MYCN is challenging, future therapeutic strategies could disrupt specific lncRNA-protein interactions to reduce MYCN levels.

## Additional files

Additional file 1: **Figure S1.** Relative genomic positions of *MYCNOS-01*, *MYCNOS-02* and *MYCN* and protein-coding potential of *MYCNOS-01*. (A) Diagram illustrating relative positions of *MYCNOS-01*, *MYCNOS-02* and *MYCN* exons. Modified from UCSC Genome Browser. (B) Predicted sequence for three forward reading frames of *MYCNOS-01*. (B) Table of results for ATGpr analysis of *MYCNOS-01*. The reliability score indicates the likelihood a start codon is in the correct context for translation initiation, with a score closer to one indicating increased likelihood. (PDF 594 kb)

Additional file 2: **Figure S2.** Correlation of *MYCNOS* transcripts and *MYCN* transcript expression in RMS measured by qRT-PCR. (A) Correlation between *MYCNOS-01* and *MYCN* measured by qRT-PCR in 80 RMS patient samples (*R* = 0.281, *p* = 0.012) and (B) correlation between *MYCNOS-02* and *MYCN* in 53 of these patient samples (*R* = 0.308, *p* = 0.025). (C) Correlation between *MYCNOS-01* and *MYCN* measured by qRT-PCR in 18 RMS cell lines (*R* = 0.821, *p* < 0.0001) and (D) correlation between *MYCNOS-02* and *MYCN* in 14 of these cell lines (*R* = 0.980, *p* < 0.0001). *MYCN*-amplified cases or cell lines are depicted with circles. RMS-01 and RH30 are indicated specifically. (E) Expression of *MYCNOS-01*, *MYCNOS-02* and *MYCN* transcripts measured by qRT-PCR in the cell lines used in experiments; RMS-01, RH30, KELLY and SY5Y. Expression relative to normal cells. (PDF 50 kb)

Additional file 3: **Figure S3.** Effect of *MYCNOS-01* knockdown on MYCN protein expression in RMS and NB measured by immunofluorescence. (A) Immunofluorescence staining of MYCN (red) with DAPI shown in blue after *MYCNOS-01* knockdown for 72 h with three siRNAs in RMS-01. (B) Quantification of MYCN staining intensity from (A) relative to total number of cells and normalised to non-targeting control. (C) qRT-PCR detecting *MYCNOS-01* transcript level matching experiment shown in (A). (D)-(F) in KELLY as for (A)-(C). Data representative of three repeats. Statistical analysis relative to non-targeting control. NT = non-targeting. (PDF 74664 kb)

Additional file 4: **Figure S4.** Effect of *MYCNOS-01* overexpression on *MYCN* transcript and protein expression. qRT-PCR detecting *MYCNOS-01* transcript level after 72 h transient transfection with two *MYCNOS-01* overexpressing vectors or empty vector control in (A) RMS-01 and (D) KELLY. Corresponding *MYCN* transcript level shown in (B) RMS-01 and (E) KELLY. Expression relative to empty vector control. MYCN protein levels after *MYCNOS-01* overexpression for 72 h shown in (C) RMS-01 and (F) KELLY. GAPDH used as loading control. Densitometry values shown above each blot normalised to GAPDH and relative to empty vector control. EV = empty vector. (PDF 3518 kb)

Additional file 5: **Figure S5.** Effect of *MYCNOS-01* knockdown on MYCN protein stability. (A) RMS-01 cells treated with three *MYCNOS-01* siRNAs for 48 h including 4 h treatment with DMSO or MG132. Densitometry values shown above each blot normalised to GAPDH and relative to NT control for each condition. Blots representative of experiments run in triplicate. (B) *MYCNOS-01* transcript expression after *MYCNOS-01* knockdown for 24 h measured by qRT-PCR. NT = non-targeting control. (PDF 584 kb)

Additional file 6: **Figure S6.** Effect of *MYCNOS-02* knockdown on *MYCN* transcript and protein expression in RMS and NB. qRT-PCR detecting *MYCNOS-02* transcript level after *MYCNOS-02* knockdown with three siRNAs in (A) RMS-01 and (D) KELLY. Corresponding *MYCN* transcript level shown in (B) RMS-01 and (E) KELLY. Expression relative to NT control. Western blots showing MYCN protein levels after *MYCNOS-02* knockdown with three siRNAs shown in (C) RMS-01 and (F) KELLY. GAPDH used as loading control. Densitometry values shown above each blot normalised to GAPDH and relative to NT control. Data representative of 3 repeats. NT = non-targeting control. Relative expression of transcripts in these cell lines are indicated in Additional file 2: Figure S2E. (PDF 3709 kb)

Additional file 7: **Figure S7.** Effect of *MYCN* knockdown on *MYCNOS-02* transcript expression in RMS and NB. (A) qRT-PCR and (B) Western blot for MYCN, and (C) qRT-PCR for *MYCNOS-02* after treatment with three pooled *MYCN* siRNAs for 72 h in RMS-01. (D)-(F) in KELLY as for (A)-(C). Graphs and Western blots representative of three repeats. NT = non-targeting control. (PDF 4860 kb)

Additional file 8: **Figure S8.** Knockdown of *MYCNOS-02* inhibits cell viability. (Ai) MTS assay showing cell viability of RMS-01 over 96 h after transfection with three *MYCNOS-02* siRNAs relative to NT siRNA. (Bi) KELLY as in (Ai). Corresponding qRT-PCR of *MYCNOS-02* (ii) at 24 h shown below line graph. Data representative of two repeats. Statistical analysis relative to non-targeting control. NT = non-targeting. (PDF 82 kb)

Additional file 9: **Figure S9.** Knockdown of *MYCNOS-01* does not induce apoptosis. (A) Caspase 3/7 signalling intensity in RMS-01 after transfection with three *MYCNOS-01* siRNAs or three pooled *MYCN* siRNAs for 96 h relative to cell viability and normalised to NT siRNA. (B) Western blot of total and cleaved PARP in RMS-01 after transfection with three *MYCNOS-01* siRNAs or three pooled *MYCN* siRNAs. (C, D) in KELLY, (E, F) in RH30 and (G, H) in SY5Y as for (A, B). Data representative of three repeats. NT = non-targeting, tPARP = total PARP, cPARP = cleaved PARP. (PDF 5703 kb)

Additional file 10: **Figure S10.** Knockdown of *MYCNOS-02* does induce apoptosis. (A) Caspase 3/7 signalling intensity in RMS-01 after transfection with three *MYCNOS-02* siRNAs for 96 h relative to cell viability and normalised to NT siRNA. (B) Western blot of total and cleaved PARP in RMS-01 after transfection with three *MYCNOS-02* siRNAs. (C, D) in KELLY as for (A, B). Data representative of two repeats. NT = non-targeting, tPARP = total PARP, cPARP = cleaved PARP. (PDF 3672 kb)

Additional file 11: **Figure S11.** Knockdown of *MYCNOS-01* does not affect cell cycle progression. (A) Flow cytometry 96 h post-transfection with three *MYCNOS-01* siRNAs or three pooled *MYCN* siRNAs in RMS-01, (B) KELLY, (C) RH30, and (D) SY5Y. Graphs show an average of three repeats. NT = non-targeting. (PDF 111 kb)

## Abbreviations

lncRNA: long non-coding RNA
NB: Neuroblastoma
RMS: Rhabdomyosarcoma
