# Supplementary figures and images for: The long non-coding RNA MYCNOS-01 regulates MYCN protein levels and affects growth of MYCN-amplified rhabdomyosarcoma and neuroblastoma cells
Source: BMC Cancer. 2018 Feb 21;18:217. doi: 10.1186/s12885-018-4129-8 (PMC5822637; doi:10.1186/s12885-018-4129-8)

Figure S1

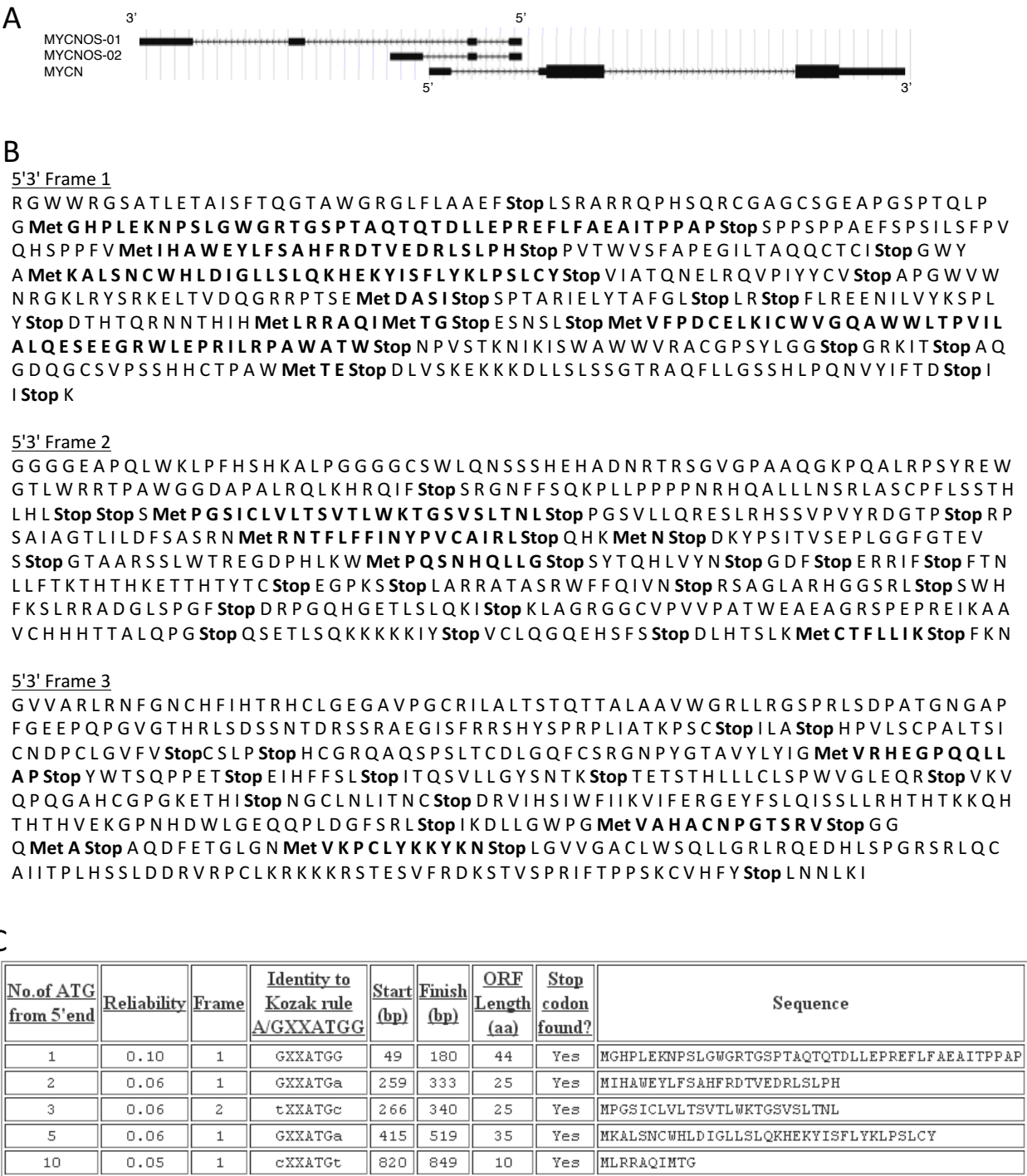

Supplement: Supplementary file 1 — Figure S1. Relative genomic positions of MYCNOS-01, MYCNOS-02 and MYCN and protein-coding potential of MYCNOS-01. (A) Diagram illustrating relative positions of MYCNOS-01, MYCNOS-02 and MYCN exons. Modified from UCSC Genome Browser. (B) Predicted sequence for three forward reading frames of MYCNOS-01. (B) Table of results for ATGpr analysis of MYCNOS-01. The reliability score indicates the likelihood a start codon is in the correct context for translation initiation, with a score closer to one indicating increased likelihood. (PDF 594 kb) [file 12885_2018_4129_MOESM1_ESM.pdf]

Figure S2

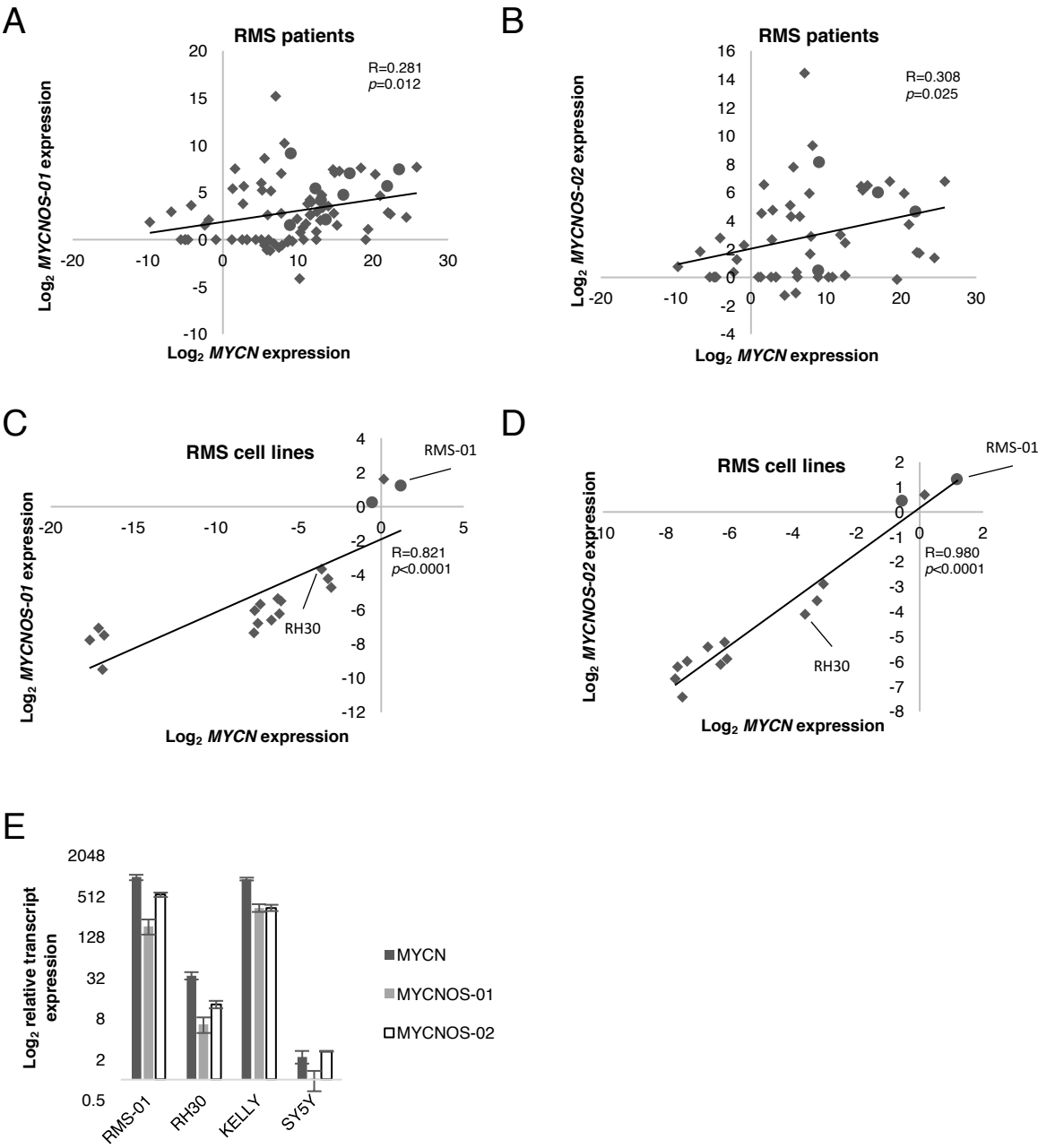

Supplement: Supplementary file 2 — Figure S2. Correlation of MYCNOS transcripts and MYCN transcript expression in RMS measured by qRT-PCR. (A) Correlation between MYCNOS-01 and MYCN measured by qRT-PCR in 80 RMS patient samples (R = 0.281, p = 0.012) and (B) correlation between MYCNOS-02 and MYCN in 53 of these patient samples (R = 0.308, p = 0.025). (C) Correlation between MYCNOS-01 and MYCN measured by qRT-PCR in 18 RMS cell lines (R = 0.821, p < 0.0001) and (D) correlation between MYCNOS-02 and MYCN in 14 of these cell lines (R = 0.980, p < 0.0001). MYCN-amplified cases or cell lines are depicted with circles. RMS-01 and RH30 are indicated specifically. (E) Expression of MYCNOS-01, MYCNOS-02 and MYCN transcripts measured by qRT-PCR in the cell lines used in experiments; RMS-01, RH30, KELLY and SY5Y. Expression relative to normal cells. (PDF 50 kb) [file 12885_2018_4129_MOESM2_ESM.pdf]

Figure S3

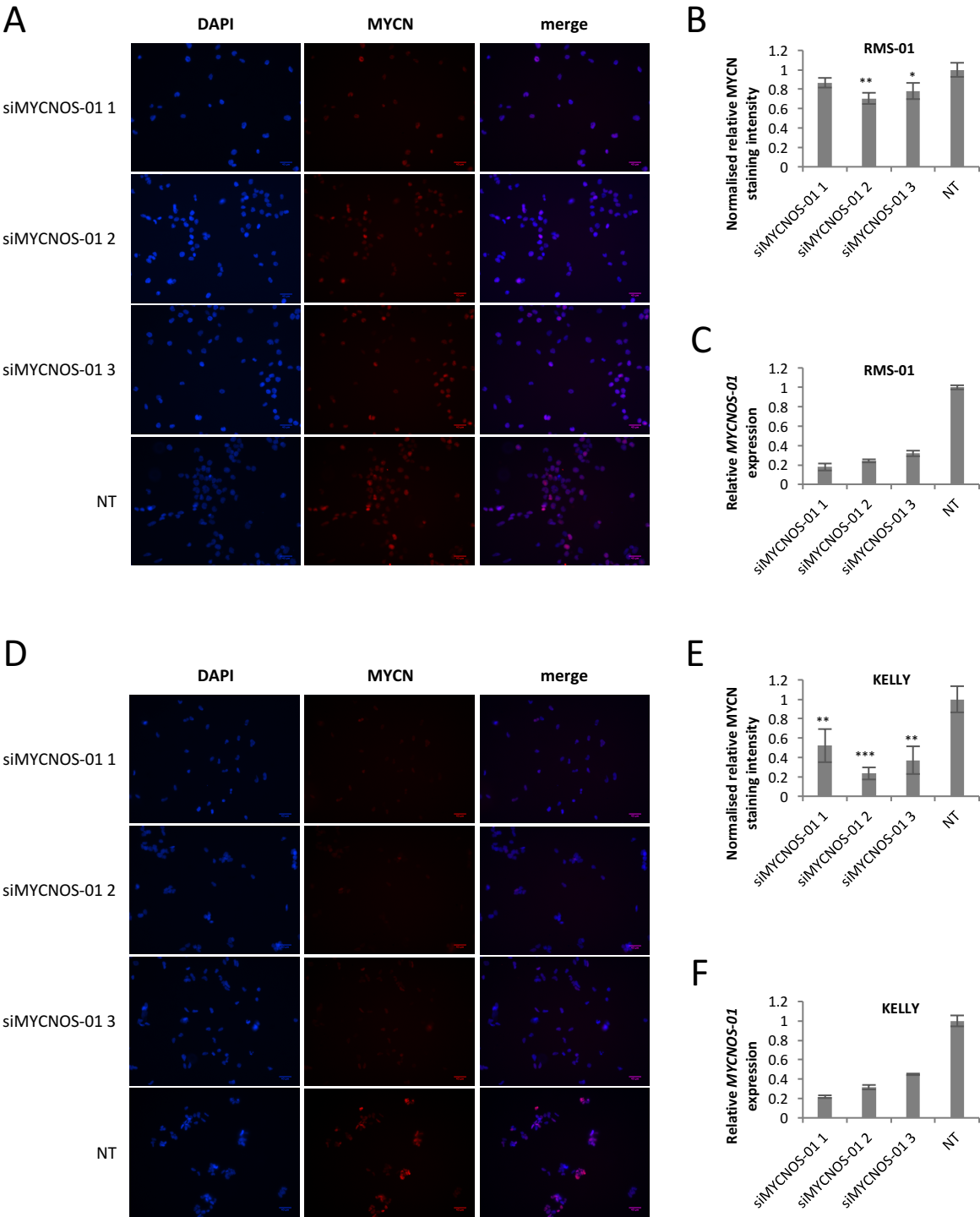

Supplement: Supplementary file 3 — Figure S3. Effect of MYCNOS-01 knockdown on MYCN protein expression in RMS and NB measured by immunofluorescence. (A) Immunofluorescence staining of MYCN (red) with DAPI shown in blue after MYCNOS-01 knockdown for 72 h with three siRNAs in RMS-01. (B) Quantification of MYCN staining intensity from (A) relative to total number of cells and normalised to non-targeting control. (C) qRT-PCR detecting MYCNOS-01 transcript level matching experiment shown in (A). (D)-(F) in KELLY as for (A)-(C). Data representative of three repeats. Statistical analysis relative to non-targeting control. NT = non-targeting. (PDF 74664 kb) [file 12885_2018_4129_MOESM3_ESM.pdf]

Figure S4

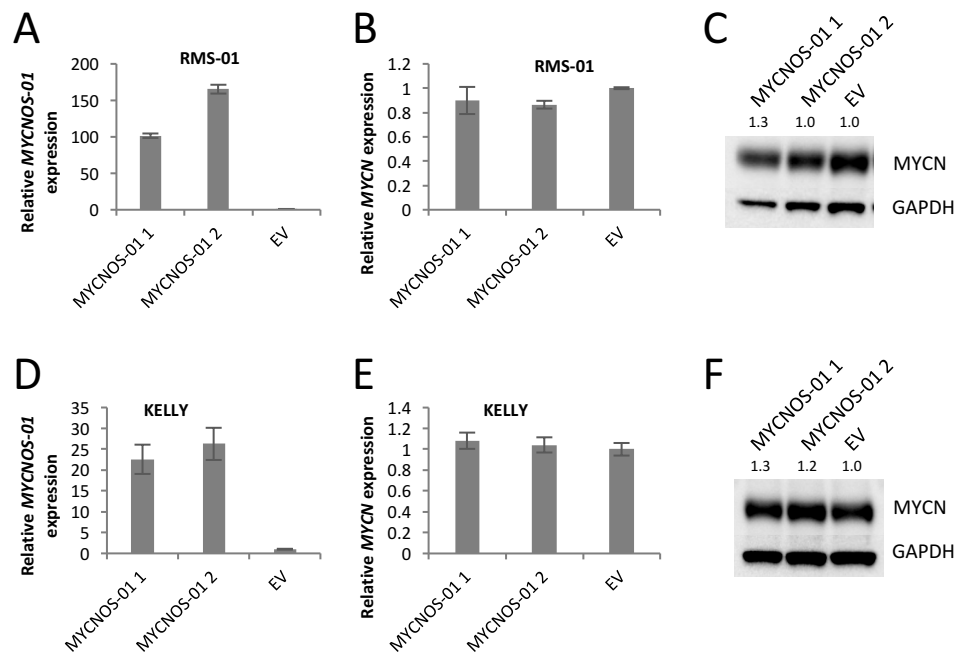

Supplement: Supplementary file 4 — Figure S4. Effect of MYCNOS-01 overexpression on MYCN transcript and protein expression. qRT-PCR detecting MYCNOS-01 transcript level after 72 h transient transfection with two MYCNOS-01 overexpressing vectors or empty vector control in (A) RMS-01 and (D) KELLY. Corresponding MYCN transcript level shown in (B) RMS-01 and (E) KELLY. Expression relative to empty vector control. MYCN protein levels after MYCNOS-01 overexpression for 72 h shown in (C) RMS-01 and (F) KELLY. GAPDH used as loading control. Densitometry values shown above each blot normalised to GAPDH and relative to empty vector control. EV = empty vector. (PDF 3518 kb) [file 12885_2018_4129_MOESM4_ESM.pdf]

Figure S5

A

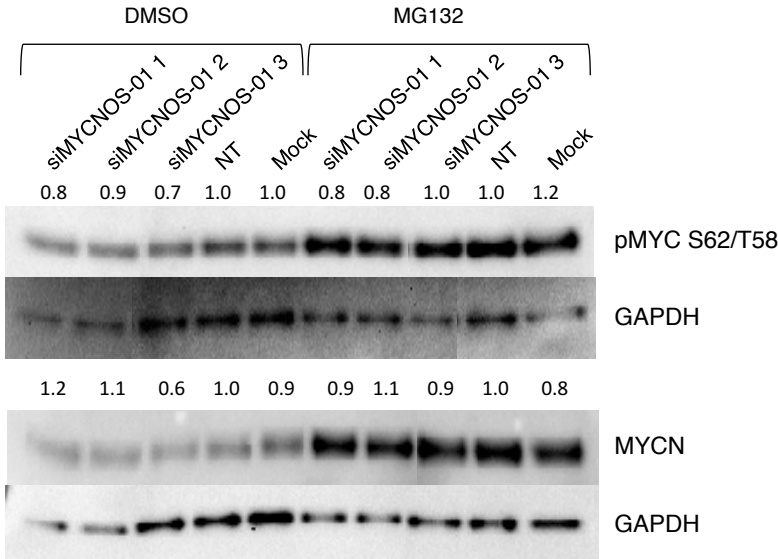

B

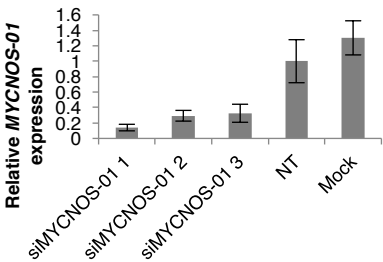

Supplement: Supplementary file 5 — Figure S5. Effect of MYCNOS-01 knockdown on MYCN protein stability. (A) RMS-01 cells treated with three MYCNOS-01 siRNAs for 48 h including 4 h treatment with DMSO or MG132. Densitometry values shown above each blot normalised to GAPDH and relative to NT control for each condition. Blots representative of experiments run in triplicate. (B) MYCNOS-01 transcript expression after MYCNOS-01 knockdown for 24 h measured by qRT-PCR. NT = non-targeting control. (PDF 584 kb) [file 12885_2018_4129_MOESM5_ESM.pdf]

Figure S6

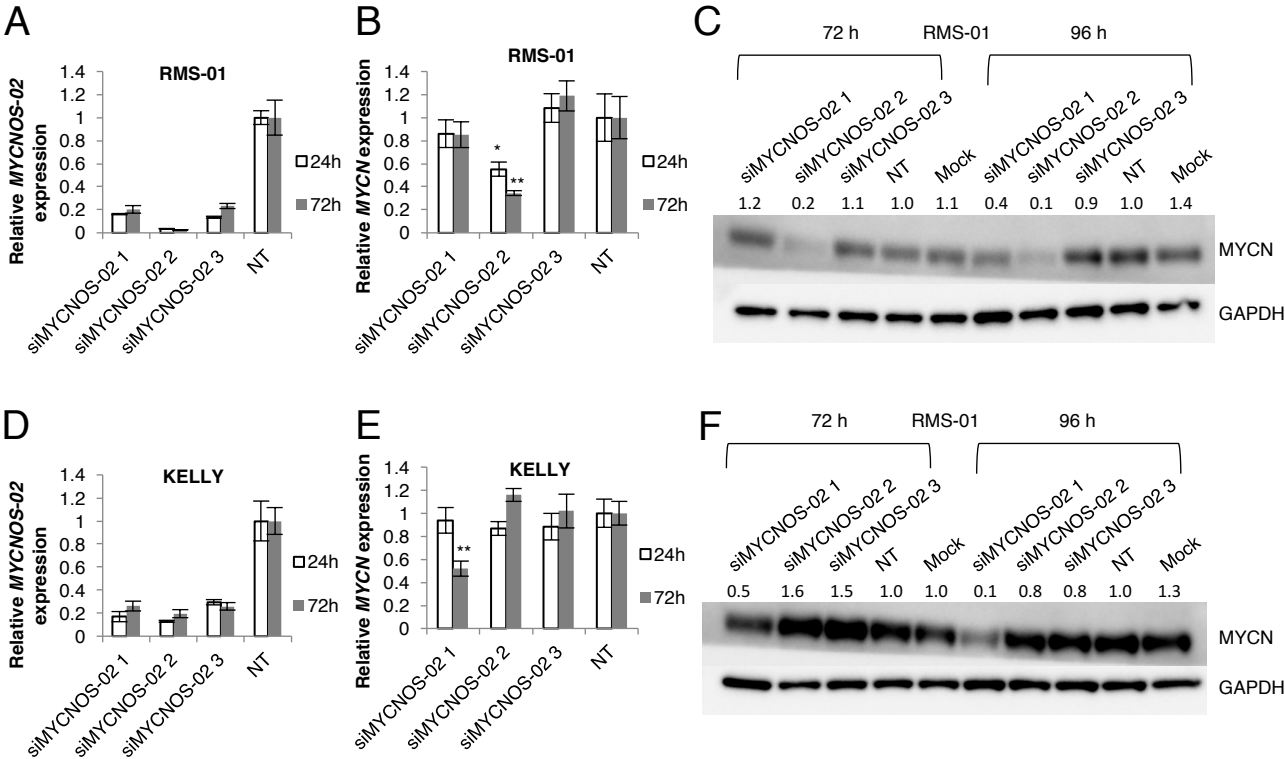

Supplement: Supplementary file 6 — Figure S6. Effect of MYCNOS-02 knockdown on MYCN transcript and protein expression in RMS and NB. qRT-PCR detecting MYCNOS-02 transcript level after MYCNOS-02 knockdown with three siRNAs in (A) RMS-01 and (D) KELLY. Corresponding MYCN transcript level shown in (B) RMS-01 and (E) KELLY. Expression relative to NT control. Western blots showing MYCN protein levels after MYCNOS-02 knockdown with three siRNAs shown in (C) RMS-01 and (F) KELLY. GAPDH used as loading control. Densitometry values shown above each blot normalised to GAPDH and relative to NT control. Data representative of 3 repeats. NT = non-targeting control. Relative expression of transcripts in these cell lines are indicated in Additional file 2: Figure S2E. (PDF 3709 kb) [file 12885_2018_4129_MOESM6_ESM.pdf]

Figure S7

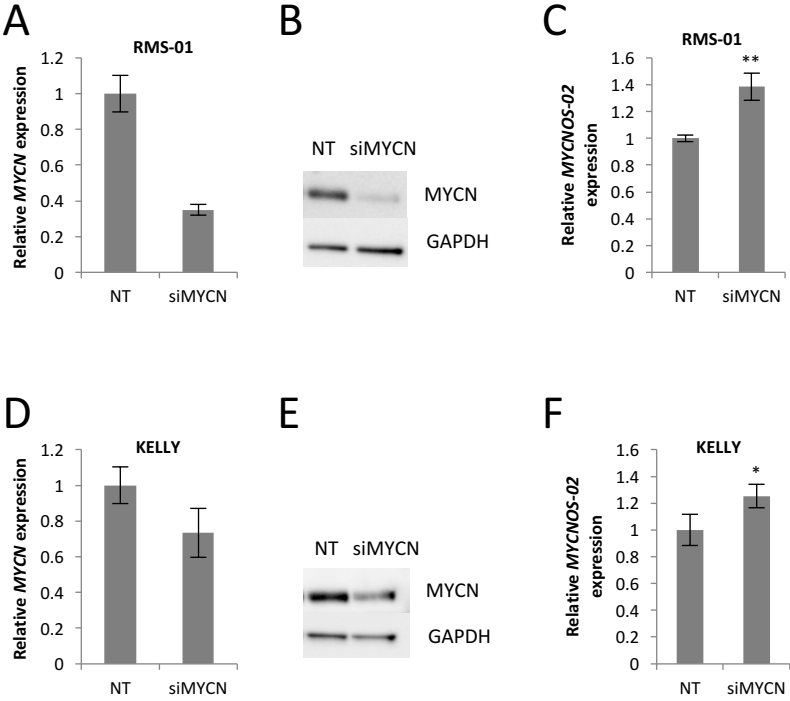

Supplement: Supplementary file 7 — Figure S7. Effect of MYCN knockdown on MYCNOS-02 transcript expression in RMS and NB. (A) qRT-PCR and (B) Western blot for MYCN, and (C) qRT-PCR for MYCNOS-02 after treatment with three pooled MYCN siRNAs for 72 h in RMS-01. (D)-(F) in KELLY as for (A)-(C). Graphs and Western blots representative of three repeats. NT = non-targeting control. (PDF 4860 kb) [file 12885_2018_4129_MOESM7_ESM.pdf]

Figure S8

A(i)

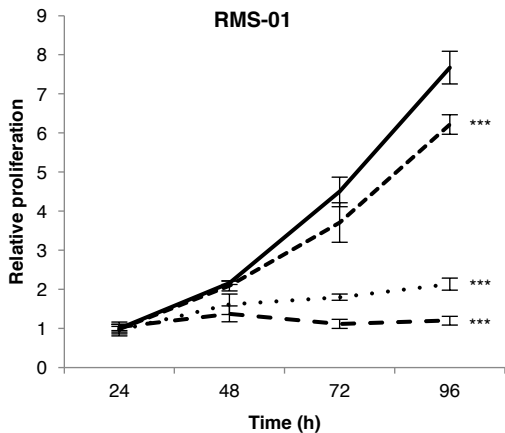

B(i)

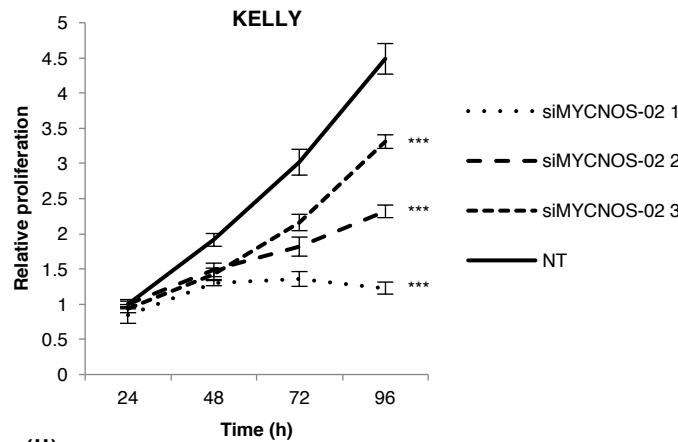

(ii)

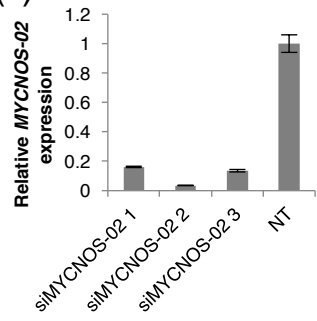

(ii)

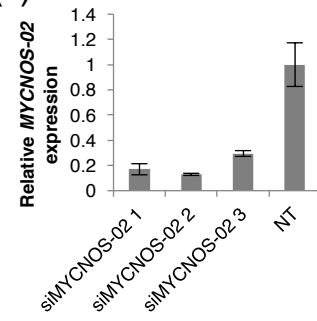

Supplement: Supplementary file 8 — Figure S8. Knockdown of MYCNOS-02 inhibits cell viability. (Ai) MTS assay showing cell viability of RMS-01 over 96 h after transfection with three MYCNOS-02 siRNAs relative to NT siRNA. (Bi) KELLY as in (Ai). Corresponding qRT-PCR of MYCNOS-02 (ii) at 24 h shown below line graph. Data representative of two repeats. Statistical analysis relative to non-targeting control. NT = non-targeting. (PDF 82 kb) [file 12885_2018_4129_MOESM8_ESM.pdf]

Figure S9

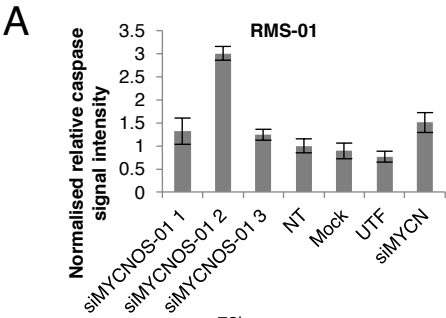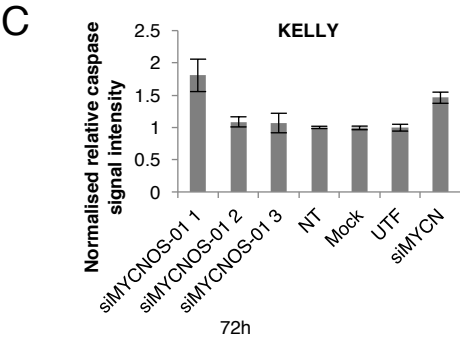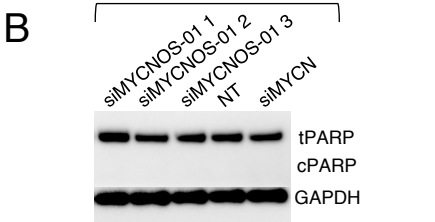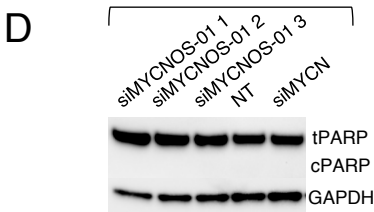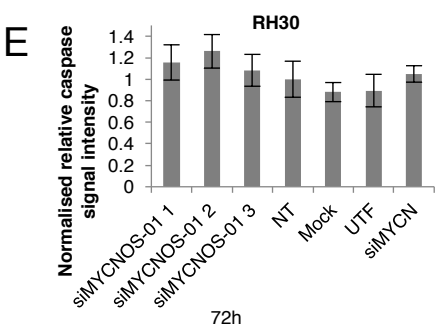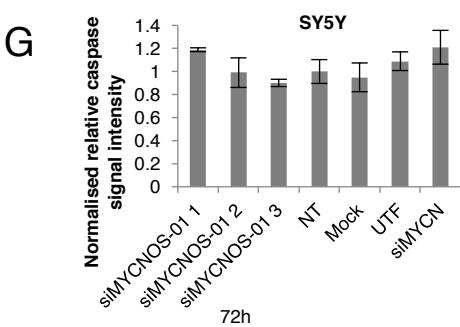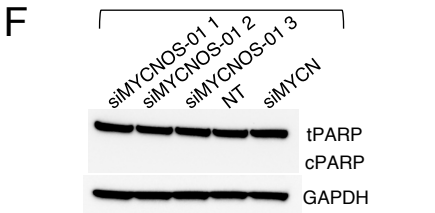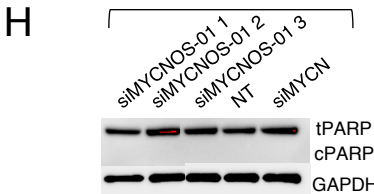

Supplement: Supplementary file 9 — Figure S9. Knockdown of MYCNOS-01 does not induce apoptosis. (A) Caspase 3/7 signalling intensity in RMS-01 after transfection with three MYCNOS-01 siRNAs or three pooled MYCN siRNAs for 96 h relative to cell viability and normalised to NT siRNA. (B) Western blot of total and cleaved PARP in RMS-01 after transfection with three MYCNOS-01 siRNAs or three pooled MYCN siRNAs. (C, D) in KELLY, (E, F) in RH30 and (G, H) in SY5Y as for (A, B). Data representative of three repeats. NT = non-targeting, tPARP = total PARP, cPARP = cleaved PARP. (PDF 5703 kb) [file 12885_2018_4129_MOESM9_ESM.pdf]

Figure S10

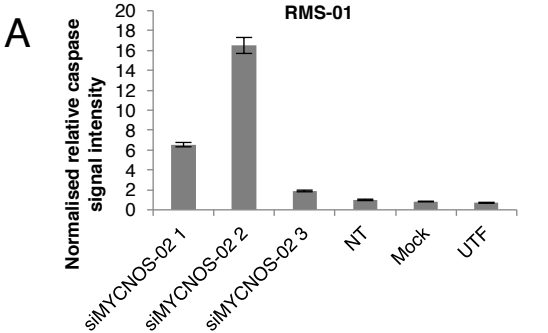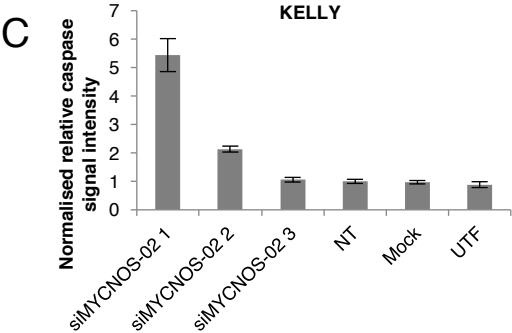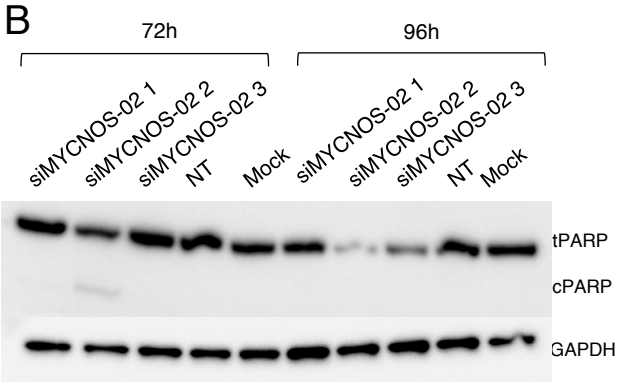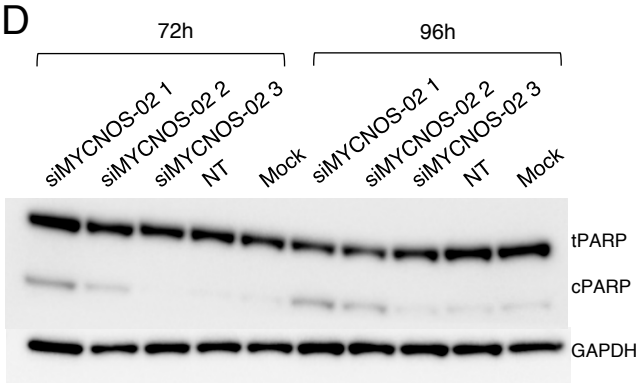

Supplement: Supplementary file 10 — Figure S10. Knockdown of MYCNOS-02 does induce apoptosis. (A) Caspase 3/7 signalling intensity in RMS-01 after transfection with three MYCNOS-02 siRNAs for 96 h relative to cell viability and normalised to NT siRNA. (B) Western blot of total and cleaved PARP in RMS-01 after transfection with three MYCNOS-02 siRNAs. (C, D) in KELLY as for (A, B). Data representative of two repeats. NT = non-targeting, tPARP = total PARP, cPARP = cleaved PARP. (PDF 3672 kb) [file 12885_2018_4129_MOESM10_ESM.pdf]

Figure S11

A

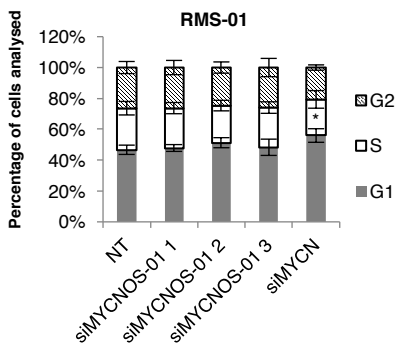

B

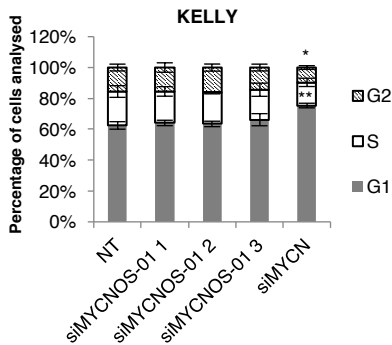

C

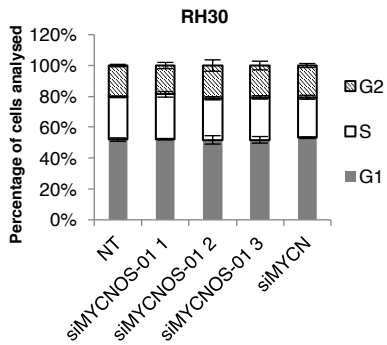

D

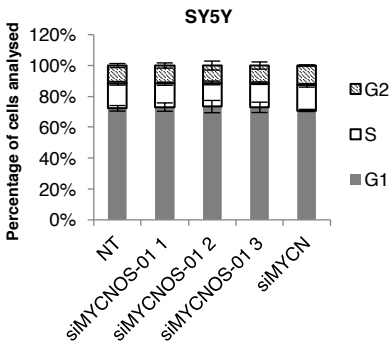

Supplement: Supplementary file 11 — Figure S11. Knockdown of MYCNOS-01 does not affect cell cycle progression. (A) Flow cytometry 96 h post-transfection with three MYCNOS-01 siRNAs or three pooled MYCN siRNAs in RMS-01, (B) KELLY, (C) RH30, and (D) SY5Y. Graphs show an average of three repeats. NT = non-targeting. (PDF 111 kb) [file 12885_2018_4129_MOESM11_ESM.pdf]
